# On the Skill of Balancing While Riding a Bicycle

**DOI:** 10.1371/journal.pone.0149340

**Published:** 2016-02-24

**Authors:** Stephen M. Cain, James A. Ashton-Miller, Noel C. Perkins

**Affiliations:** 1 Department of Mechanical Engineering, The University of Michigan, Ann Arbor, Michigan, United States of America; 2 Department of Biomedical Engineering, The University of Michigan, Ann Arbor, Michigan, United States of America; 3 School of Kinesiology, The University of Michigan, Ann Arbor, Michigan, United States of America; Purdue University, UNITED STATES

## Abstract

Humans have ridden bicycles for over 200 years, yet there are no continuous measures of how skill differs between novice and expert. To address this knowledge gap, we measured the dynamics of human bicycle riding in 14 subjects, half of whom were skilled and half were novice. Each subject rode an instrumented bicycle on training rollers at speeds ranging from 1 to 7 m/s. Steer angle and rate, steer torque, bicycle speed, and bicycle roll angle and rate were measured and steering power calculated. A force platform beneath the roller assembly measured the net force and moment that the bicycle, rider and rollers exerted on the floor, enabling calculations of the lateral positions of the system centers of mass and pressure. Balance performance was quantified by cross-correlating the lateral positions of the centers of mass and pressure. The results show that all riders exhibited similar balance performance at the slowest speed. However at higher speeds, the skilled riders achieved superior balance performance by employing more rider lean control (quantified by cross-correlating rider lean angle and bicycle roll angle) and less steer control (quantified by cross-correlating steer rate and bicycle roll rate) than did novice riders. Skilled riders also used smaller steering control input with less variation (measured by average positive steering power and standard deviations of steer angle and rate) and less rider lean angle variation (measured by the standard deviation of the rider lean angle) independent of speed. We conclude that the reduction in balance control input by skilled riders is not due to reduced balance demands but rather to more effective use of lean control to guide the center of mass via center of pressure movements.

## Introduction

Humans have ridden bicycles since the early 1800’s. Nevertheless, the control strategies humans employ to balance a bicycle while riding are not well understood. The Whipple bicycle model is the simplest model that predicts the self-stability of the bicycle alone [1, 2]; however, the behavior of an uncontrolled bicycle provides only partial understanding of a bicycle controlled by a human. Identifying the types of control that humans use and understanding the differences between skilled and novice riders would promote two advances. The former would provide researchers with metrics to evaluate rider skill and human-on-bicycle stability, while the latter would provide bicycle designers with tools to objectively evaluate the balance performance of any bicycle/rider pair.

Bicycle riding skill or performance has previously been assessed by instructing subjects to ride around a prescribed course or to perform a prescribed task. The time to complete the course or task, and the number of errors committed, were proposed as performance measures [3, 4]. While useful for answering the questions posed in [3, 4], these outcome measures are task specific and do not translate to new tasks. Furthermore, these outcome measures do not provide continuous monitoring of skill, which is important for understanding how riders respond to internal or external perturbations.

Studies of motorcycle riding suggest that body lean and steering torque discriminate riders of different skill levels. In measuring motorcycle rider behavior during a lane change maneuver, Rice [5] found that rider skill was distinguished by different phasing between body lean and steering torque. Similarly, Prem [6] observed that novice riders also used different lean and steering torque relative to expert riders in an evasive maneuver. In particular, novice riders exhibited a strong coupling between leaning motion and steering inputs, whereas expert riders controlled leaning and steering independently. Similar to the aforementioned studies on bicycles [3, 4], Prem [6] employed outcome measures in skill tests to differentiate rider ability.

The ability to balance a bicycle is necessary to successfully complete any riding task, yet no methods have previously been proposed to quantify that balance skill. By contrast, balance skill methods for human standing balance and postural control are well established and include monitoring the location of the center of pressure (COP) relative to the center of mass (COM) [7] which are highly correlated [8]. For example, using an inverted pendulum to model standing balance, Winter [7] demonstrated that the (COP—COM) location difference signal is directly related to the horizontal acceleration of the COM and could be considered to be the error signal detected by the balance control system. The assumption is that the goal of the balance control system is to maintain an upright posture and to control postural sway. Measures of postural sway, typically quantified by COP movement metrics, are commonly utilized by clinicians to identify patients with balance disorders [9].

In the present study, our aim is to develop and test methods for evaluating human-on-bicycle control and balance. Unlike standing balance, balancing a bicycle is a highly dynamic task that requires coordination of the human subject and the bicycle. For quasi-static stance, stability requires that the projection of the COM falls within the base of support; but during more dynamic tasks, the projection of the COM can fall outside of the base of support provided momentum returns it inside the base of support [10, 11]. By extending from these studies of standing balance, we aim to reveal the relationship of COM to COP movements during bicycling.

Previous research on bicycling has explored possible control methods used by riders [12, 13], methods to quantify control [14–16], and differences between skilled and unskilled riders [17]. Theory demonstrates that lean control and steer control are effective in stabilizing a bicycle model [14–16], and both are observed experimentally with human riders [13, 18]. Lean control may alter steer torque [12], which in turn alters steer angle. Both lean control and steer control are observed by calculating cross-correlations of lean angle and steer angle/rate with bicycle roll angle/rate [15, 16]. Our pilot experiment [17] revealed that skilled cyclists steer less and use less steer power than non-skilled cyclists, suggesting that control magnitude and control variation can distinguish rider skill.

Extending from our pilot study [17], the objectives of this study are three-fold: 1) to quantify the relationship between COP and COM movements of the rider-bicycle system, 2) to reveal the types of control used by riders, and 3) to quantify the differences between skilled and novice riders. We hypothesize that 1) the lateral position of the COM will be highly correlated to the lateral position of the COP, 2) steer rate will be highly correlated to the roll rate of the bicycle, 3) rider lean will be highly correlated to bicycle roll, and 4) skilled riders will use significantly smaller steering control input and variation than novice riders.

## Methods

We tested a total of 14 subjects (4 females, 10 males; age = 26.4 ± 6.0 years, body mass = 71.1 ± 12.8 kg; mean ± standard deviation). The University of Michigan Health Sciences and Behavioral Sciences Institutional Review Board approved the study, and all subjects gave written informed consent.

**Fig 1:**
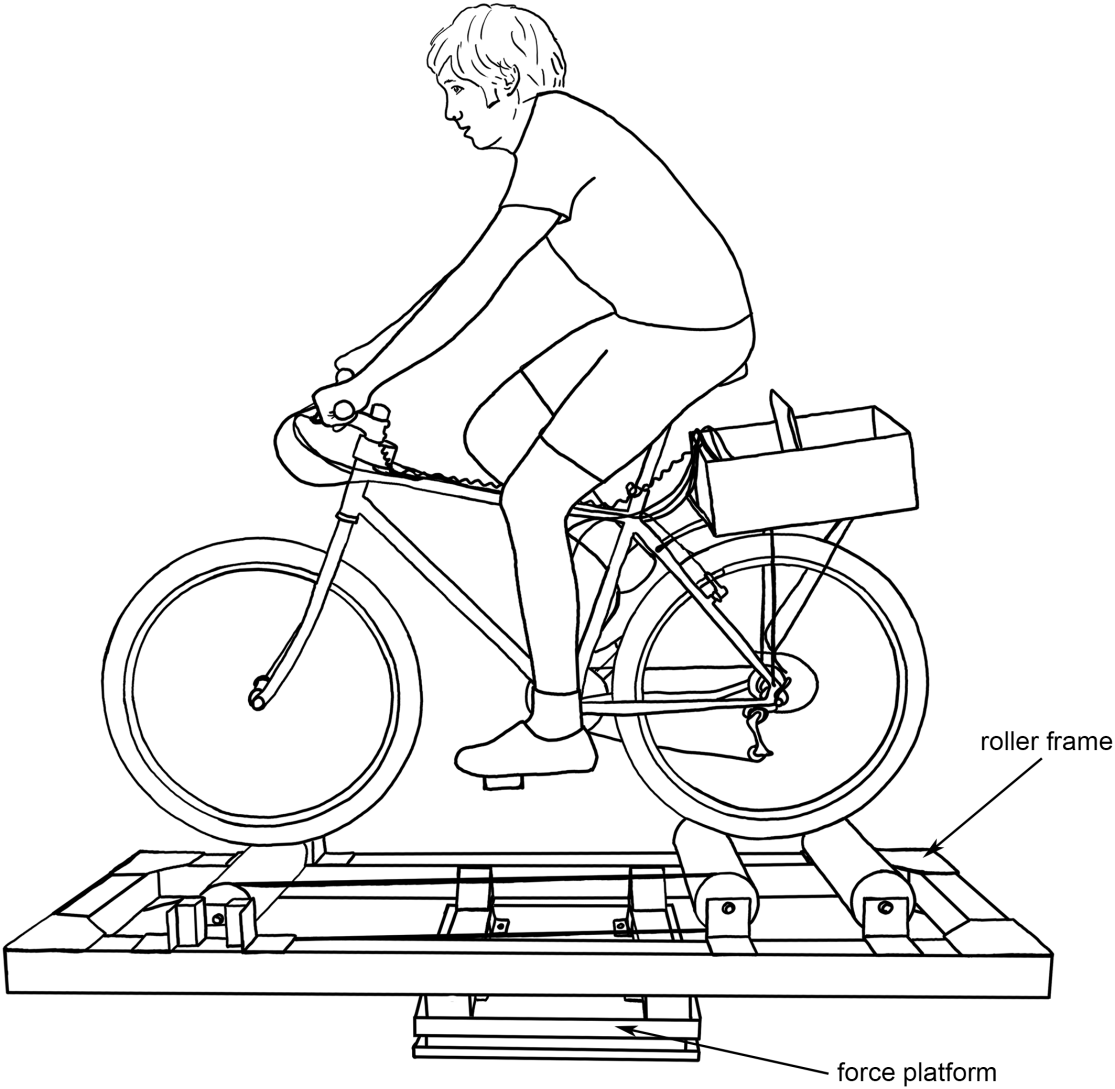
A subject riding the instrumented bicycle on the training rollers. The training rollers were bolted to a force platform via four brackets attached to the rectangular frame on which the rollers were mounted.

Seven subjects were classified as “cyclists” and seven subjects as “non-cyclists.” All cyclists identified themselves as skilled cyclists, went on regular training rides, belonged to a cycling club or team, competed several times per year, and had used rollers (Fig 1) for training indoors. The non-cyclists knew how to ride a bicycle but did so only occasionally for recreation or transportation and did not identify themselves as skilled cyclists.

To measure the dynamics of human bicycle riding, we conducted experiments indoors utilizing an instrumented bicycle [12], a motion capture system (Optotrak 3020, NDI), and training rollers mounted on a force platform (OR6-5-2000, AMTI). The instrumented bicycle [12] incorporated sensors that measured steer angle (*δ*), steer torque (*T*_*δ*_), bicycle speed (*v*), and bicycle frame roll rate ($\dot{\phi }$). In addition, we calculated steering power from steer torque and steer angular velocity. The motion capture system measured the positions of three markers attached to the bicycle frame, which were used to calculate the roll angle of the bicycle frame (*ϕ*). The force platform beneath the roller assembly measured the net force and moment that the rider-bicycle-roller system exerted on the ground. These reactions were later used to calculate the lateral positions of the bicycle-rider COP and COM. Utilizing the measured and calculated quantities, we examine standard deviations of signals and cross-correlations between signals to reveal differences in rider skill.

Rollers, which constrain the bicycle in the fore-aft direction but allow free lateral movement, require a bicycle rider to maintain balance by pedaling, steering and leaning, as one would outdoors. The dynamics of a bicycle on rollers are similar to that of a bicycle overground [19] with the following distinctions. The cylindrical surface of rollers introduces: 1) a slightly different shape for the tire contact patch, 2) a geometric constraint between the front wheel and front roller as the bicycle steers and yaws, and 3) a small moment on the rear wheel from the two rollers it contacts [19]. Because the bicycle on rollers is stationary in the fore/aft direction, riders do not experience the same visual cues as they do riding outdoors.

Compared to bicycle riding on a treadmill [13, 18, 20], rollers offer distinct advantages for investigating human-bicycle dynamics. Rollers weigh less than a treadmill, which is advantageous when also mounting to a force platform that places strict limits on ground reactions. In addition, riding on rollers is a safer task than riding on a treadmill. Riding a bicycle on rollers is also more challenging than riding overground, and therefore may be particularly useful in eliciting differences between skilled riders (cyclists) and less skilled riders (non-cyclists).

### Experimental protocol

Each subject rode under five experimental conditions distinguished by pedaling cadence, established via a metronome, and bicycle ‘speed’ controlled via gearing (the method for measuring speed is given in Instrumentation). The five conditions were executed in the following order: 1) cadence 80 rpm and speed 5.08 m/s, 2) cadence 80 rpm and speed 7.19 m/s, 3) cadence 80 rpm and speed 6.98 m/s, 4) cadence 80 rpm and speed 2.58 m/s, and 5) cadence 40 rpm and speed 1.29 m/s. Each subject rode for a minimum of 2 minutes in each condition until s/he could ride for at least 30 seconds without touching a static support surface with hand or foot. A platform placed over the rollers allowed subjects to safely dismount the bicycle and a safety railing beside the rollers allowed subjects to support themselves during trials if needed. One cyclist and five non-cyclists were unable to successfully ride on the rollers at the slowest speed defined in condition 5, and instead rode with a cadence of 50 rpm and a speed of 1.61 m/s. We instructed riders to ride on the rollers for at least 30 seconds without support and to match their pedaling rate to the beat of the metronome as closely as possible. We did not instruct riders on how to ride the rollers or what type of control to use.

Prior to the data collection, we allowed subjects to practice riding the instrumented bicycle on the rollers as long as they needed to become comfortable. During this time, we also ensured that the seat height was set properly for each subject. Cyclists generally needed only a few minutes to familiarize themselves with the instrumented bicycles. Non-cyclists, all of whom had no experience riding on rollers, generally needed 10 to 15 minutes to become familiar with the bicycle and to learn how to ride on rollers.

### Instrumentation

The instrumented bicycle is described in detail in [12]. It was a standard geometry rigid (no suspension) mountain bike equipped with slick tires. A torque sensor (Transducer Techniques SWS-20) integrated into the fork steering tube measured the steer torque applied by the bicycle rider (*T*_*δ*_) with a range of ±7.5 Nm and a resolution of 0.005 Nm. An optical encoder disk (US Digital HUBDISK-2-1800-1125-I) and encoder module (US Digital EM1-2-1800) captured the steer angle of the bicycle (*δ*) with a resolution of 0.1 degrees. Numerical differentiation of the steer angle yields steer angle velocity ($\dot{\delta }$), which when multiplied by steer torque yields the instantaneous power used by the rider to steer the bicycle. Integration of steering power yields the steering work which is further decomposed into positive and negative work components. We obtained the bicycle speed (*v*) by using the measured circumference of the front wheel and wheel revolutions recorded using a magnetic reed switch and magnet (Cateye 169–9772 and 169–9691). A three-axis accelerometer (Analog Devices ADXL335) and three single-axis angular rate gyros (Murata ENC-03M) measured the acceleration of the bicycle frame with a range and resolution of ±29.43 and 0.067m/s^2^, respectively, and the angular velocity of the bicycle frame with a range and resolution of ±300 deg/s and 3.04 deg/s, respectively. We calculated the bicycle roll rate ($\dot{\phi }$) by resolving the angular velocity measured by the frame-mounted angular rate gyros into a bicycle-fixed frame, with one axis parallel to the roll axis of the bicycle [12]. We sampled all signals at 1 kHz except the steer angle, which was sampled at 200 Hz. Steer torque, steer angle, steer velocity, and roll rate were low-pass filtered at 10 Hz.

A motion capture system measured the positions of three markers rigidly attached to the headtube of the instrumented bicycle at a sampling rate of 750 Hz. We applied a low-pass filter with a cut-off frequency of 10 Hz to the position data. A marker triad allowed us to calculate roll, pitch, and yaw of the bicycle frame relative to an inertial frame. For this study, only the roll angle was required. We calculated the roll angle of the bicycle as:

$$\phi ={\text{sin}}^{-1}\left(\frac{z_{3}-z_{2}}{\left|\vec{r_{23}}\right|}\right)-\phi_{0}$$ (1)

where *z*_2_ and *z*_3_ are the (vertical) z-coordinates of markers 2 and 3 (the pair of markers furthest apart) relative to the inertial frame, respectively, $\left|\vec{r_{23}}\right|$ is the magnitude of the position vector from marker 2 to marker 3, and *ϕ*_0_ is the known angle that $\vec{r_{23}}$ makes with the horizontal plane when the roll angle of the bicycle is zero. The camera system measured the position of the markers with a maximum RMS error of 0.15 mm. This RMS error in position translates to the maximum RMS error of 0.09 degrees for the bicycle roll angle.

We designed and constructed custom rollers (Fig 1) to be bolted to the top plate of a force platform (OR6-5-2000, Advanced Mechanical Technology, Inc.). The custom rollers consist of commercially available drums and belt (Kreitler Challenger 4.5, Mountain Racing Products) and a frame built from aluminum T-slotted framing (15 Series T-slotted aluminum and joining plates, 80/20 Inc.). The drums selected for the custom rollers were the largest diameter commercially available. We chose larger drums as they produce less rolling resistance (making it easier for subjects to pedal) and are easier to balance on than smaller drums [21]. The frame allowed the rollers to be mounted to the force platform (Fig 1) so that the ground reactions acting on the bicycle-rider-roller system could be measured.

The design of the custom rollers allowed adjustment of the horizontal and vertical positions of the front roller to achieve the same front wheel contact as a bicycle riding on level ground. Specifically, the vertical adjustment enabled the bicycle to be level and the horizontal adjustment enabled the front wheel to contact the roller directly beneath the font axle as it does when riding on level ground.

### Data analysis

**Fig 2:**
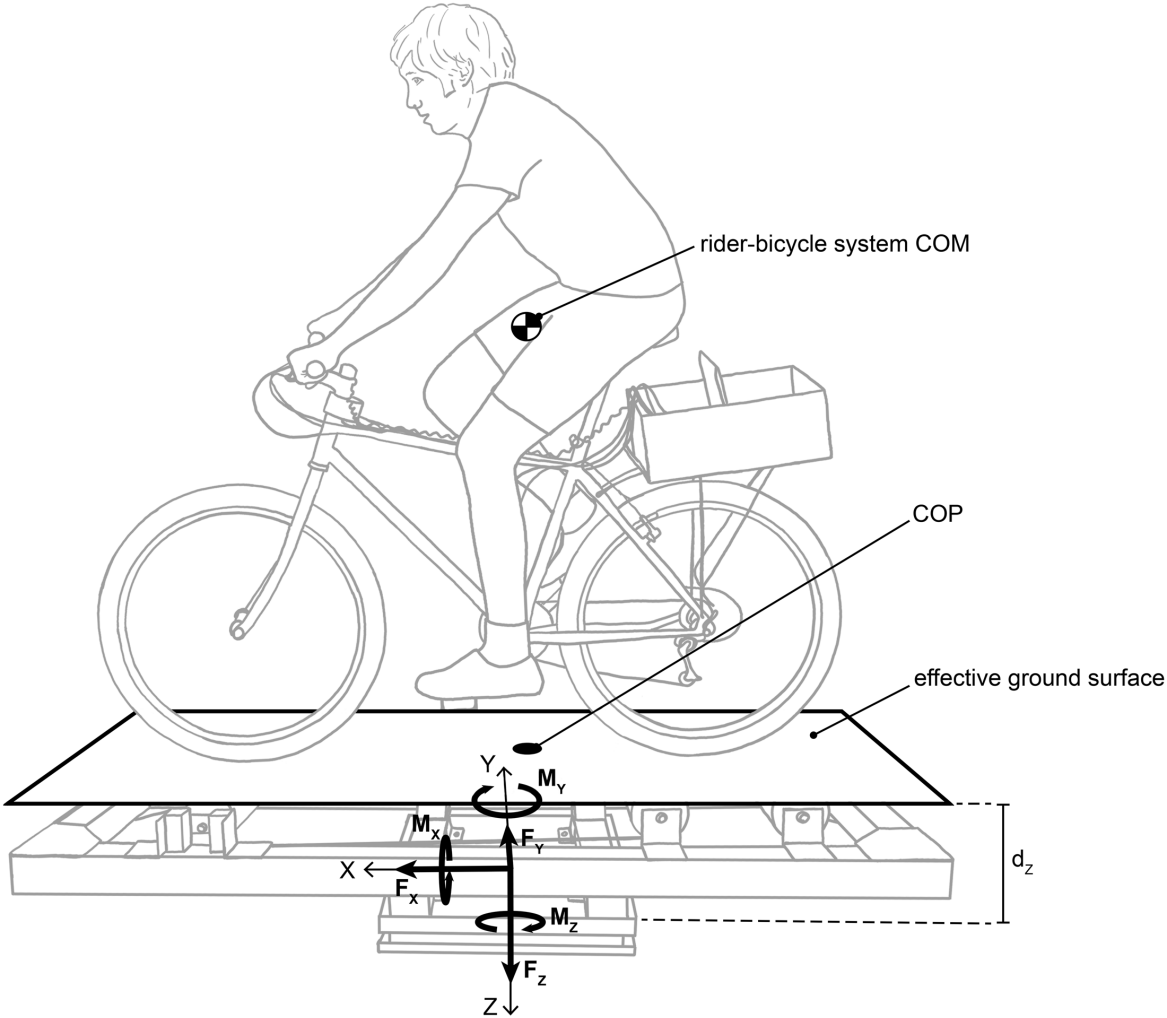
Force and moment measurements from the force platform were used to calculate the rider-bicycle system COM and COP locations in the lateral or y-direction. The ground reaction forces (*F*_*x*_, *F*_*y*_, *F*_*z*_) and moments (*M*_*x*_, *M*_*y*_, *M*_*z*_) were measured by the force platform beneath the rollers. The bicycle rides on an effective ground surface located a distance *d*_*z*_ above the force platform.

We utilized the force and moment measurements from the force platform to calculate the rider-bicycle system centers of pressure (COP) and mass (COM) locations in the lateral or y-direction (Fig 2). We sampled all six channels from the force platform at 1 kHz. After bolting the rollers to the force platform, we zeroed all signals. In addition, we also included about 10 seconds of data at the beginning of each trial so that the signal DC offsets could be identified and removed during post-processing. We applied a low-pass filter with a cut-off frequency of 10 Hz to the measured forces and moments.

The center of pressure location was calculated as follows:

$$x_{COP}=\frac{-\left(M_{y}+F_{x}d_{z}\right)}{F_{z}}$$ (2)

$$y_{COP}=\frac{\left(M_{x}-F_{y}d_{z}\right)}{F_{z}}$$ (3)

where *x*_*cop*_ and *y*_*cop*_ are the coordinates of the center of pressure, *F*_*x*_, *F*_*y*_, and *F*_*z*_ are forces measured by the force platform, *M*_*x*_ and *M*_*y*_ are moments measured by the force platform, and *d*_*z*_ is the vertical distance from the origin of the force platform coordinate system to the plane above representing the surface that the bicycle rides on (Fig 2). The surface is defined by a horizontal plane tangent to the top surface of front roller. Eqs (2) and (3) assume that the force platform and rollers represent a single rigid body and that there is no couple applied about the x or y axis (a pure couple about the x or y axis would require attachment of the bicycle to the rollers). We utilize the lateral position of the center of pressure *y*_*cop*_ in the subsequent analysis.

To calculate the lateral position of the rider-bicycle system center of mass (*y*_*COM*_), we implemented a zero-point-to-zero-point integration technique [22, 23] that has been validated for evaluating postural control [22, 24, 25]. Double integration of the bicycle/rider lateral acceleration (*a*_*y*_) yields displacement of the rider-bicycle center of mass; however, the initial constants of integration (initial velocity and initial position) are not known. The zero-point-to-zero-point integration method is based on the assumption that when the lateral force is zero (*F*_*y*_ = 0), the horizontal position of the center of mass and the center of pressure coincide (*y*_*COM*_ = *y*_*COP*_). By integrating from one zero point to another zero point, both the initial velocity and position can be determined. The zero-point-to-zero-point integration technique is not the only method that can be used to estimate the center of mass displacement using force platform measurements; other methods utilize dynamic models of the balancing system and specific filtering techniques [26–29].

Using force platform data to estimate the center of mass position (kinetics-based method) has several advantages over using motion capture data (kinematics-based method). Kinematics-based methods rely on modeling the human body with a number of rigid body segments. By estimating the mass properties of each segment, usually using anthropometric data [30], the position of the total-body center of mass can be calculated if the location and orientation of each segment is known [8, 31]. Kinematics-based methods require using many markers so that the location and orientation of each body segment can be measured accurately as well as a sufficient number of cameras to continuously track all markers. In addition, kinematics-based methods are more sensitive than kinetics-based methods to inaccuracies in body segment parameters, especially in regard to segment lengths and head-arms-trunk parameters [25]. By choosing a kinetics-based method to calculate center of mass displacement, we avoided inaccuracies due to errors in estimated body segment properties and, in addition, simplified the experimental setup.

To our knowledge, the zero-point-to-zero-point integration technique has not been applied to study center of mass location during cycling. Therefore, we conducted four trials in which we compared the lateral location of the center of mass calculated from force platform data using the zero-point-to-zero-point integration technique with that calculated using extensive motion capture data; the results are detailed in S1 Appendix. Our results indicated that utilizing the zero-point-to-zero-point integration technique for bicycle riding yielded COM positions as good as, if not better than, the technique used for postural analysis.

**Fig 3:**
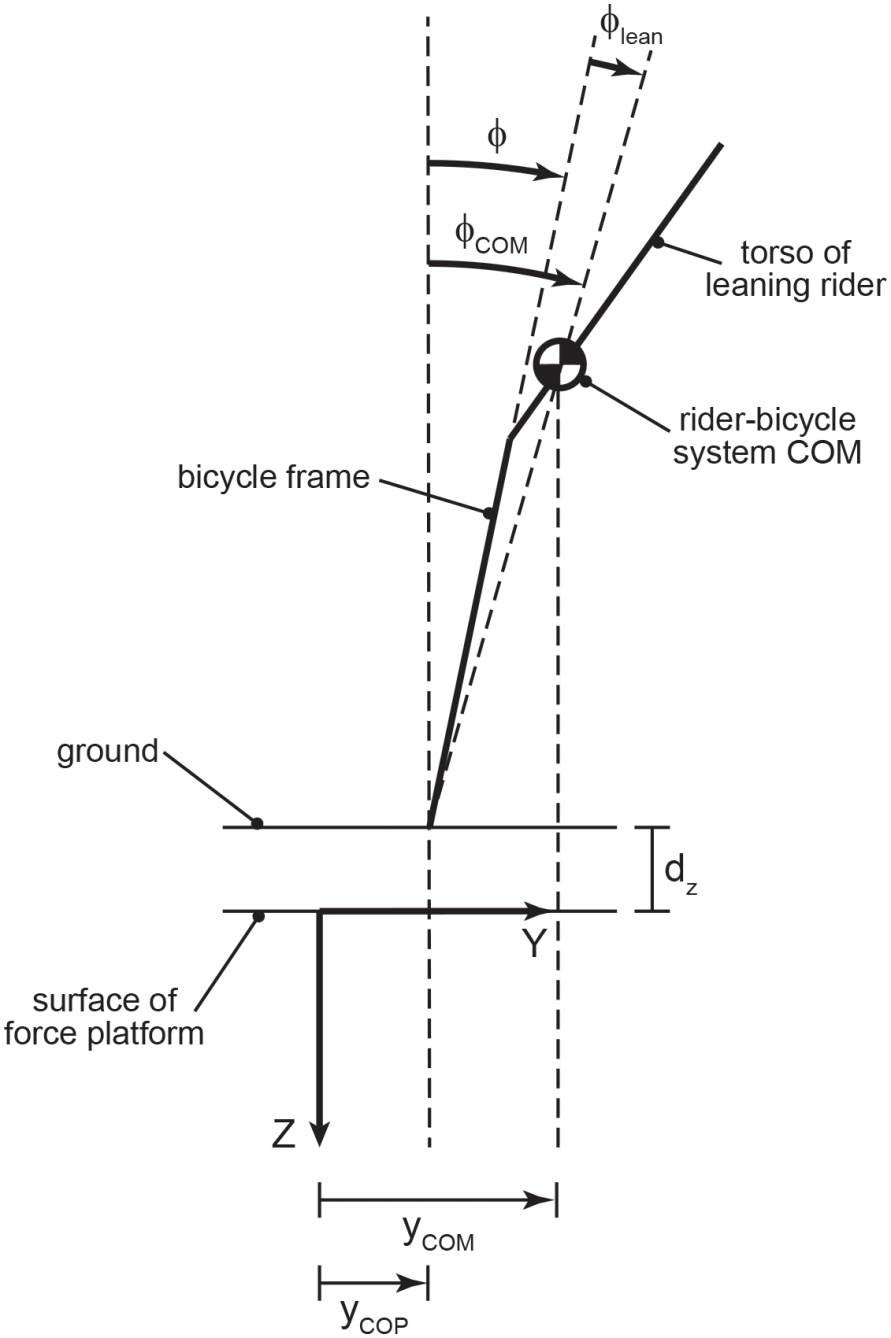
Rider lean angle as viewed from behind the rider-bicycle. The rider lean angle (*ϕ*_*lean*_) quantifies how a rider is shifting his/her COM to alter the location of the rider-bicycle COM relative to the bicycle. The arrows define the positive sense of all angles. Rider lean (*ϕ*_*lean*_) is defined as the COM roll angle (*ϕ*_*COM*_) minus the bicycle roll angle (*ϕ*).

To further explore rider control we calculated the rider lean angle (*ϕ*_*lean*_) and rider lean rate (${\dot{\phi }}_{lean}$). We define the rider lean angle as:

$$\phi_{lean}=\phi_{COM}-\phi$$ (4)

where *ϕ* is the bicycle roll angle and *ϕ*_*COM*_ is the angle formed by the line connecting the center of pressure (*y*_*COP*_) and the center of mass (*y*_*COM*_) with vertical (Fig 3). The resulting rider lean angle quantifies how a rider is shifting his/her center of mass relative to the bicycle. Note that a rider lean angle can be created in many ways, including: leaning the upper body, shifting laterally on the bicycle saddle, knee movements, arm movements, and head movements. We use the term “rider lean angle” for simplicity, as all of these motions can have the effect of moving a rider’s center of mass outside of the plane of the bicycle frame. The center of mass roll angle, *ϕ*_*COM*_, was computed via

$$\phi_{COM}={\text{sin}}^{-1}\left(\frac{y_{COM}-y_{COP}}{-z_{T}}\right)$$ (5)

where *y*_*COP*_ is the lateral position of the center of pressure, *y*_*COM*_ is the lateral position of the center of mass, and *z*_*T*_ is the location of the bicycle/rider center of mass in the z-direction when the bicycle and rider are upright, as described and as measured in [12]. The rider lean rate (${\dot{\phi }}_{lean}$) was calculated from the rider lean angle (*ϕ*_*lean*_) via numerical differentiation.

Only the last 30 seconds of each trial, when the subjects rode continuously without external support, were used for analysis. To identify periods of continuous riding, we examined plots of: vertical force measured by the force platform (*F*_*z*_), steer torque (*T*_*δ*_), and bicycle speed (*v*). We used bicycle speed to identify when a subject was pedaling. Inspection of the vertical force revealed when a subject was supported by the railing or platform (decrease in vertical force). Examination of the steer torque also identified periods that a subject used the railing for support (oscillations decrease, non-zero offset).

In order to quantify rider skill, we examined the standard deviations of signals, cross-correlations between signals, and linear relationships between cross-correlated signals. Normalized cross-correlations [32] were calculated using the *xcorr* function in the MATLAB Signal Processing Toolbox. For a given pair of signals, we squared the peak value of the normalized cross-correlation to yield the peak coefficient of determination, or *R*^2^ value, between the two signals. The *R*^2^ value provided a measure of the similarity between the pair of signals.

We used mixed linear models [33] (allowing us to account for repeated measures and unequal variances) implemented using a statistics software package (IBM SPSS Statistics) to test for significant effects. We assumed an auto regressive covariance model with an order of one and performed our statistical analyses using an alpha level of 5% (α = 0.05). The models include effects of rider type (cyclist or non-cyclist), speed, and the interaction of rider type with speed. This approach yielded two linear fits (one for each rider type) for each dependent variable. Including the effect of rider type allowed each fit to have a different y-intercept, and including the rider type × speed interaction allowed each fit to have a different slope. Therefore, significant effects of either rider type or the rider type × speed interaction indicated significant differences between cyclists and non-cyclists.

## Results and Discussion

S1 Data contains all study data required to reproduce the figures and statistical results in the following sections.

### Relationship between the center of mass and center of pressure

**Fig 4:**
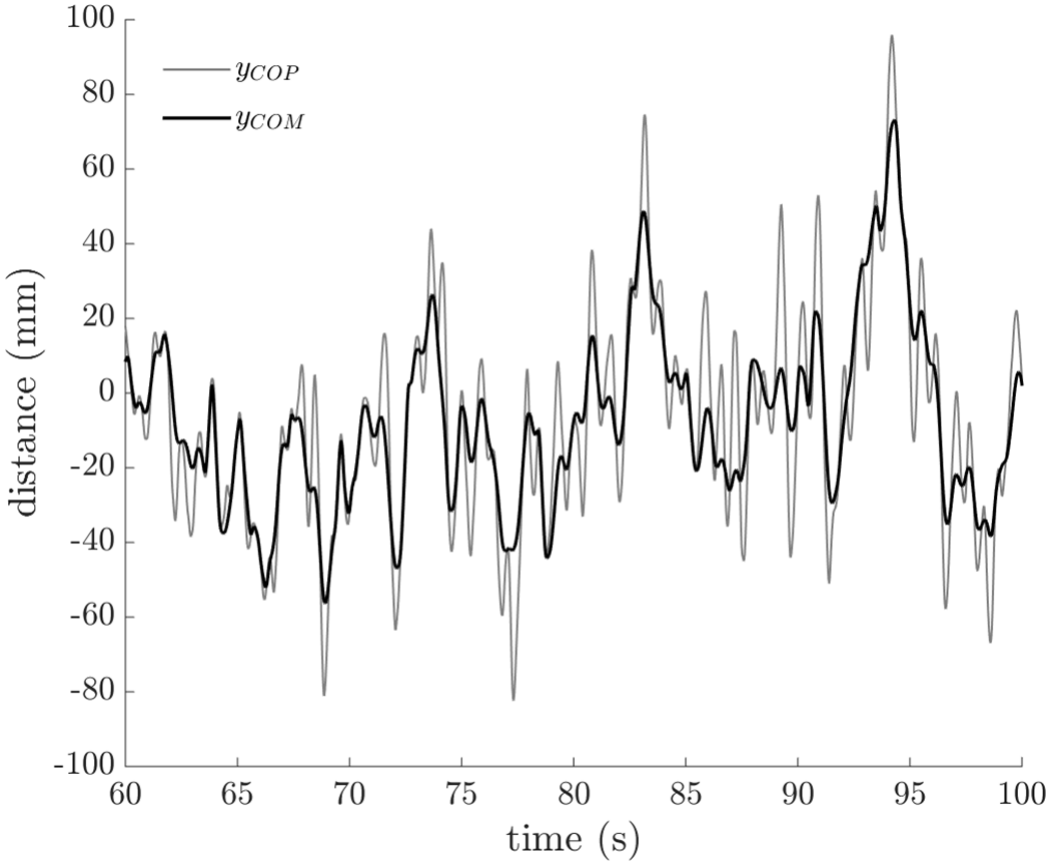
Lateral center of pressure location (*y*_*COP*_) and center of mass location (*y*_*COM*_) versus time. Data from a representative trial (non-cyclist, *v* = 7.46 m/s) demonstrates the lateral center of mass location closely tracks the lateral center of pressure location during bicycle riding.

For a perfectly balanced rider-bicycle system traveling in a straight line, we would expect *y*_*COM*_ = *y*_*COP*_. However, as in human standing, *y*_*COM*_ ≠ *y*_*COP*_ during actual bicycle riding. Instead, we expect the positions of the centers of mass and pressure co-vary, and that is what we observed (as shown in Fig 4 for a typical 40 second period).

**Fig 5:**
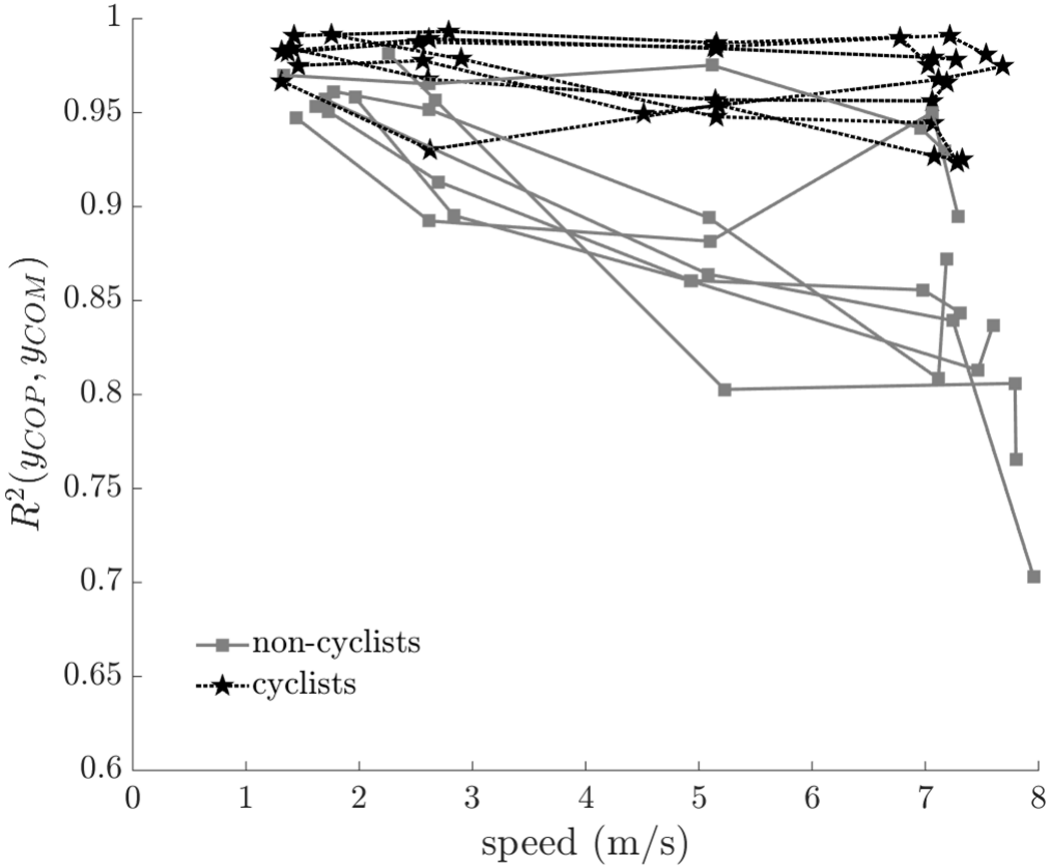
Cross-correlation of the lateral position of the center of mass (*y*_*COM*_) to the center of pressure (*y*_*COP*_) versus speed. The cross-correlation decreased significantly with increasing speed (F = 29.113, p < 0.001) and decreased significantly more with increasing speed for non-cyclists than cyclists (F = 14.843, p < 0.001). All subject data are shown; connected points indicate data from the same subject.

We quantified balance performance by calculating the cross-correlation (*R*^2^) of the center of mass location (*y*_*COM*_) to the center of pressure location (*y*_*COP*_). For a perfectly balanced bicycle, *R*^2^ would be equal to one (1.0), but for bicycle riding, we observe values less than one (Fig 5).

The lateral positions of the center of mass and center of pressure were highly correlated during bicycle riding (Fig 5). Our results did not indicate a significant effect of rider type (F = 0.041, p = 0.841), but did show significant effects for both speed (F = 29.113, p < 0.001) and the rider type × speed interaction (F = 14.843, p < 0.001). All riders demonstrated high correlation at low speeds. But as speed increased, cyclists maintained a higher correlation than non-cyclists.

### Steering

**Fig 6:**
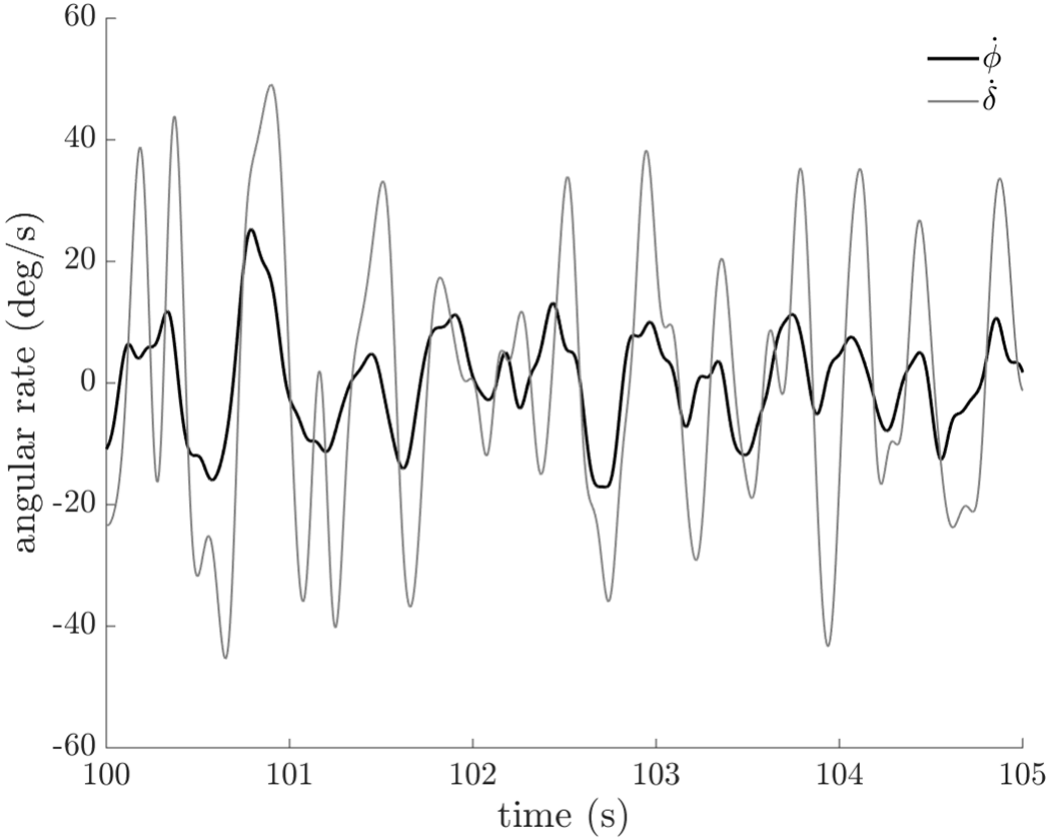
Bicycle roll rate ($\dot{\varphi }$) and steer rate ($\dot{\delta }$) versus time. Data from a representative trial (non-cyclist, *v* = 7.96 m/s) demonstrates that the steer rate ($\dot{\delta }$) lags and is correlated to the bicycle roll rate ($\dot{\phi }$) during riding.

The steer rate lagged but was correlated to the bicycle roll rate during riding (Fig 6). Similar to the methods used in [14], we quantified steer control by calculating the cross-correlations between steer rates and bicycle roll rates.

**Fig 7:**
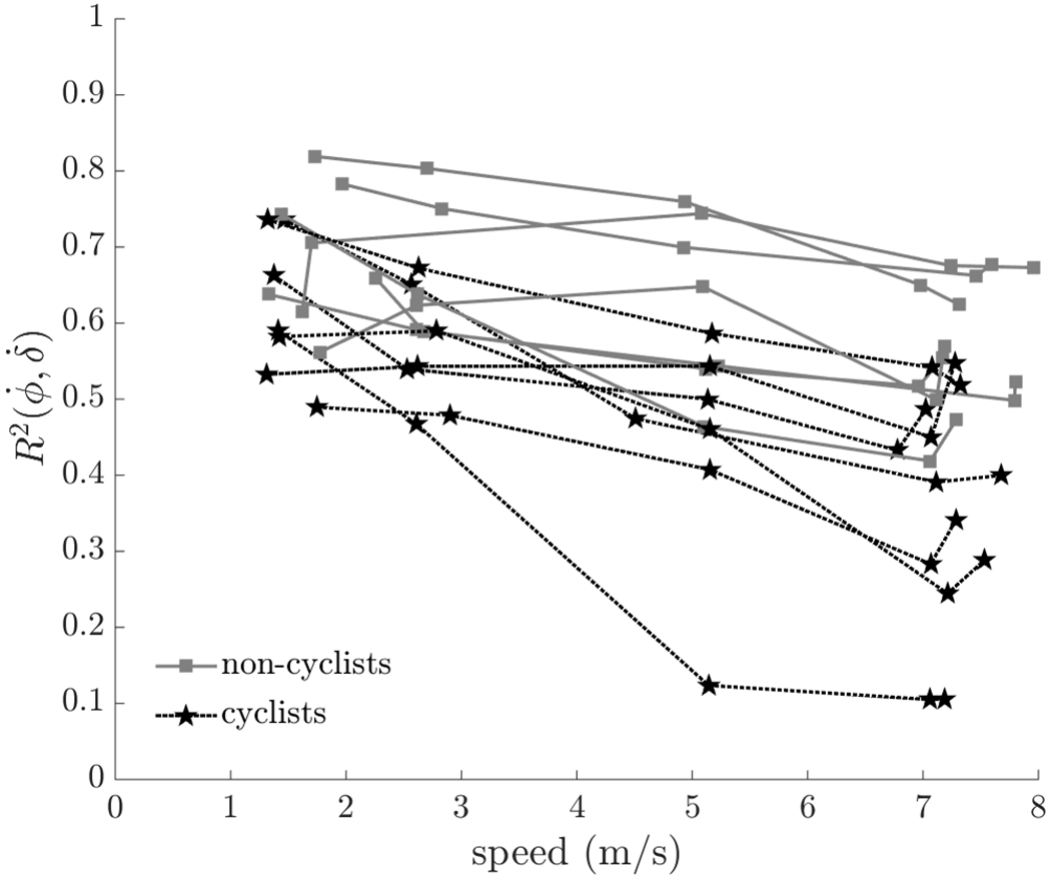
Cross-correlation of steer rate ($\dot{\delta }$) to bicycle roll rate ($\dot{\varphi }$) versus speed. The cross-correlation decreases significantly with increasing speed (F = 34.307, p < 0.001) and decreases significantly more with increasing speed for cyclists than non-cyclists (F = 4.650, p = 0.035). All subject data are shown; connected points indicate data from the same subject.

All riders demonstrated a significant correlation between steer rate and bicycle roll rate (Fig 7). The cross-correlation of steer rate to bicycle roll rate decreased significantly with increasing speed (F = 34.307, p < 0.001), more so for the cyclists than non-cyclists (F = 4.650, p = 0.035). The peak cross-correlations between steer and bicycle roll rates for the riders in this study were less than those exhibited by riders who have just learned to ride a bicycle [14]. These results suggest that cyclists and non-cyclists use steering similarly to control the bicycle when riding at low speeds, but then employ different control strategies at higher speeds.

**Fig 8:**
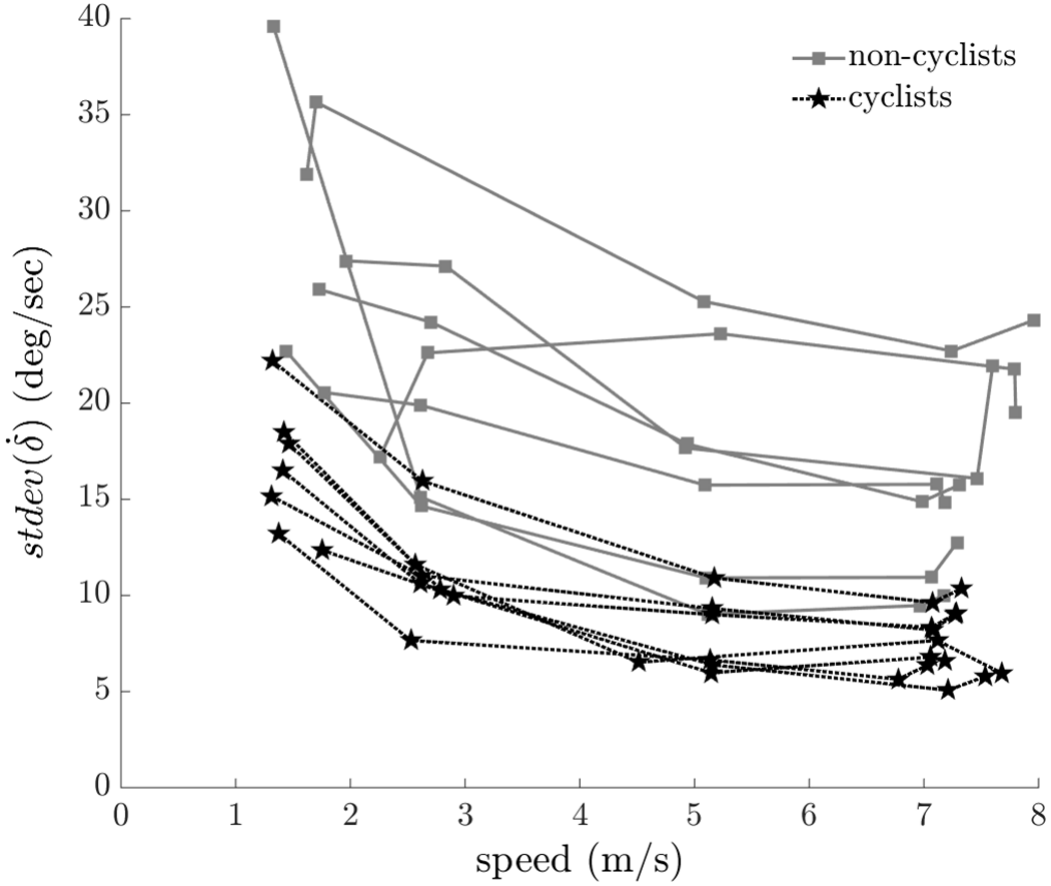
Standard deviation of steer rate ($\dot{\delta }$) versus speed. Cyclists exhibit less variation of steer rate (F = 15.121, p < 0.001) than non-cyclists. All subject data are shown; connected points indicate data from the same subject.

**Fig 9:**
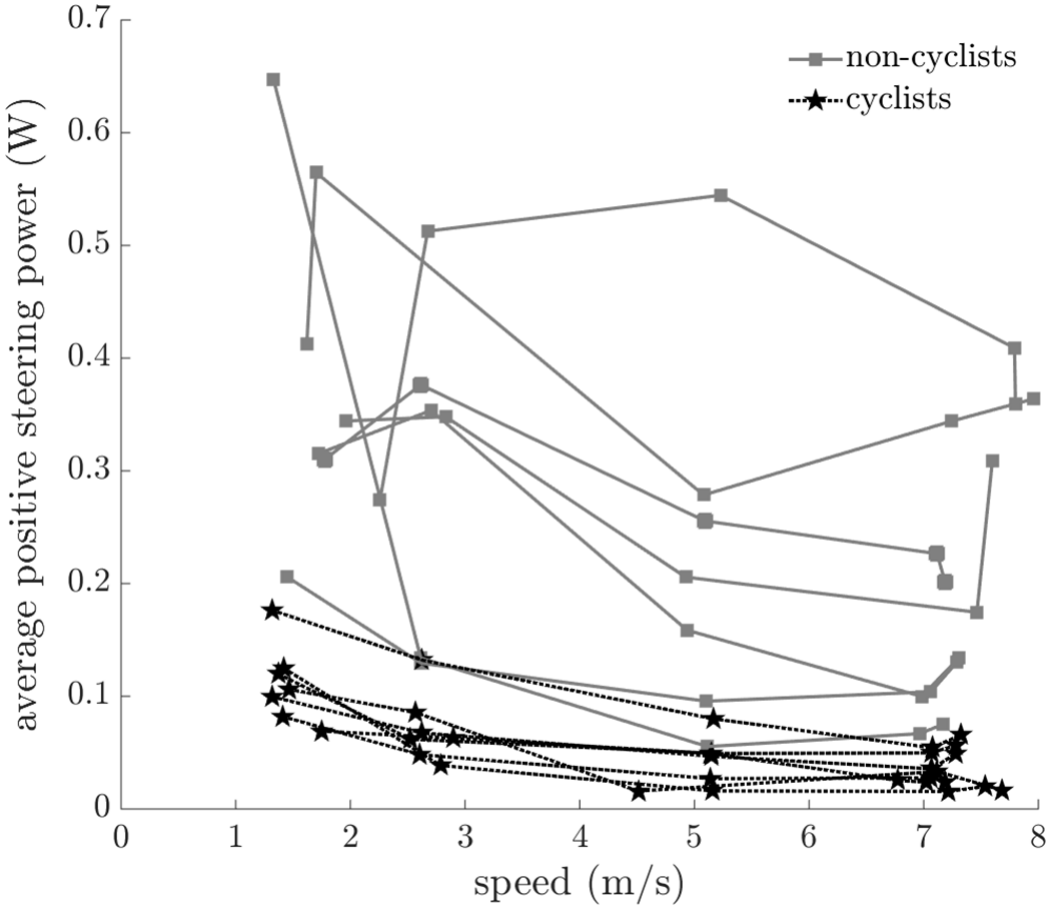
Average positive steering power versus speed. All riders developed less positive power to steer the bicycle as speed increased (F = 10.547, p = 0.002). Cyclists developed less positive power than non-cyclists (F = 19.213, p < 0.001). All subject data are shown; connected points indicate data from the same subject.

To quantify the steering control inputs, we calculated the standard deviations of the steer angle, steer rate (Fig 8), and average positive steering power (Fig 9). The standard deviations of the steer angle and steer rate and the average positive steering power decreased significantly with increasing speed for all riders. These results are consistent with subject comments that riding at higher speeds seems easier than riding at lower speeds; lower speeds required larger steering control input, as measured by both the variation of the steer angle and the amount of positive power that a rider must produce for steering. Our finding that increased speed results in decreased standard deviation of steer angle is consistent with the results of Moore et al. [13], who found that the variation of the steer angle decreased with increasing speed for a bicycle ridden on a treadmill. The cyclists exhibited less variation of steer angle (F = 13.904, p = 0.001), less variation of steer rate (F = 15.121, p < 0.001), and less positive steering power (F = 19.213, p < 0.001) than non-cyclists. The higher skill level of the cyclists enabled smaller steering control input to be used to maintain balance while riding.

### Rider lean

**Fig 10:**
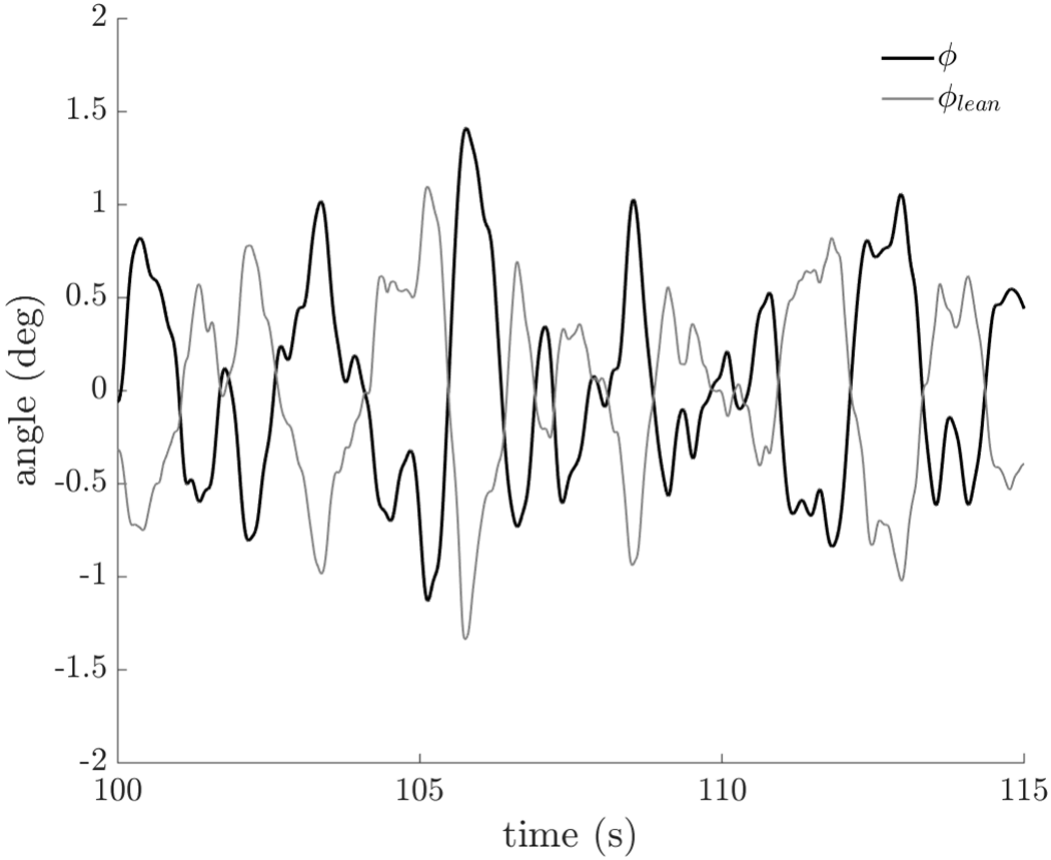
Bicycle roll angle (*ϕ*) and rider lean angle (*ϕ*_*lean*_) versus time. Data from a representative trial (cyclist, *v* = 2.526 m/s) demonstrates that rider lean (*ϕ*_*lean*_) is highly correlated, but negatively so, with bicycle roll angle (*ϕ*).

We observed that riders utilized body movements to shift the center of mass of the bicycle/rider system outside the plane of the bicycle frame and we use rider lean, (Eq 4), to quantify these movements. During riding, rider lean correlated negatively with the bicycle roll angle, as shown in Fig 10. For example, when the bicycle rolls to the left, the rider leans to the right. By leaning to the right, the rider shifts the lateral position of the center of mass of the bicycle/rider closer to the lateral position of the center of pressure. Rider lean is exaggerated and most easily observed when watching a professional road cyclist or a BMX racer rise out of the saddle to sprint; in both instances, the rider’s body remains primarily above the tire contact patch (i.e., center of pressure) as the bicycle rocks left and right beneath the rider. If rider lean (*ϕ*_*lean*_) is equal and opposite to the bicycle roll angle (*ϕ*), then the lateral position of the center of mass (*y*_*COM*_) will equal the lateral position of the center of pressure and the center of mass roll angle (*ϕ*_*COM*_) will be zero. If a rider is riding such that *y*_*COM*_ is always equal to *y*_*COP*_ while the bicycle is rolling, then the cross-correlation will equal one (1.0).

**Fig 11:**
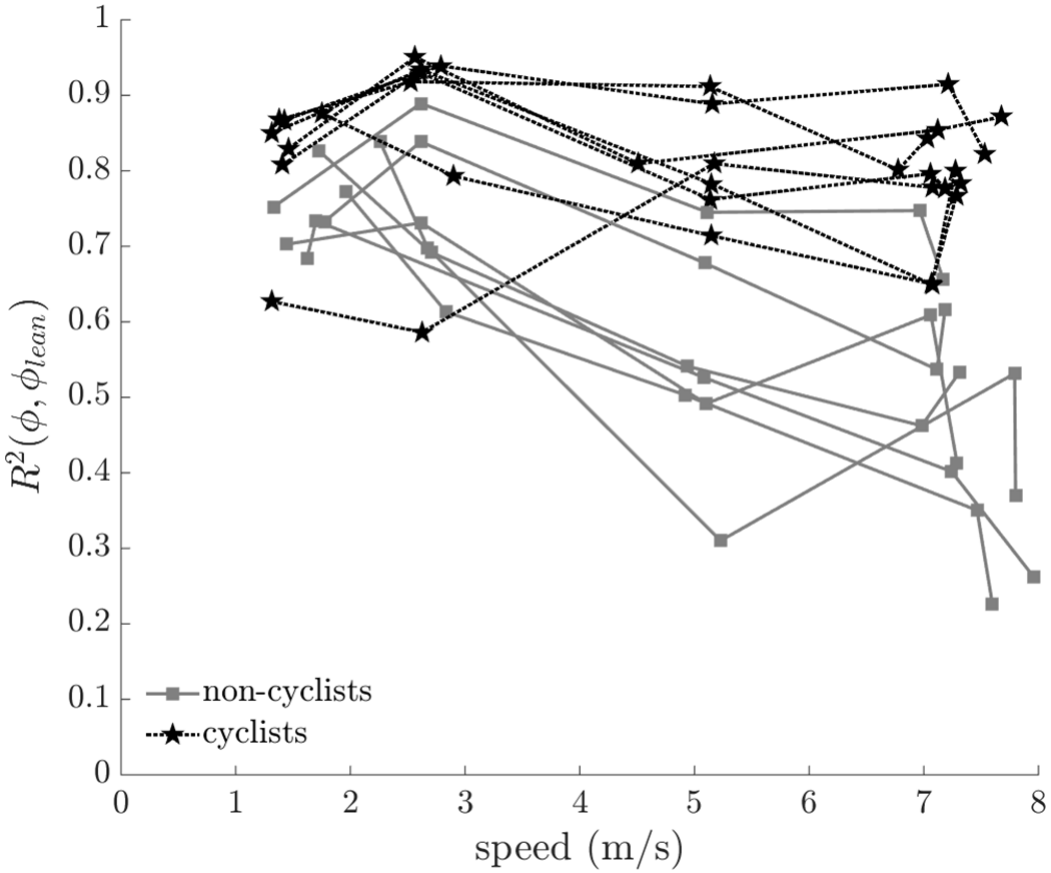
Cross-correlation of rider lean angle (*ϕ*_*lean*_) to bicycle roll angle (*ϕ*) versus speed. The cross-correlation decreases significantly with increasing speed (F = 32.948, p < 0.001) and decreases significantly more with increasing speed for non-cyclists than cyclists (F = 17.639, p < 0.001). All subject data are shown; connected points indicate data from the same subject.

We quantified rider lean control by calculating the cross-correlation of rider lean angle to bicycle roll angle. The cross-correlation of the rider lean angle to the bicycle roll angle (Fig 11) decreased with increasing speed for all riders (F = 32.948, p < 0.001) but decreased more for non-cyclists than cyclists (F = 17.639, p < 0.001). Like the findings for steer control, these results suggest that cyclists and non-cyclists use similar rider lean strategies at low speeds, but employ different strategies at higher speeds.

**Fig 12:**
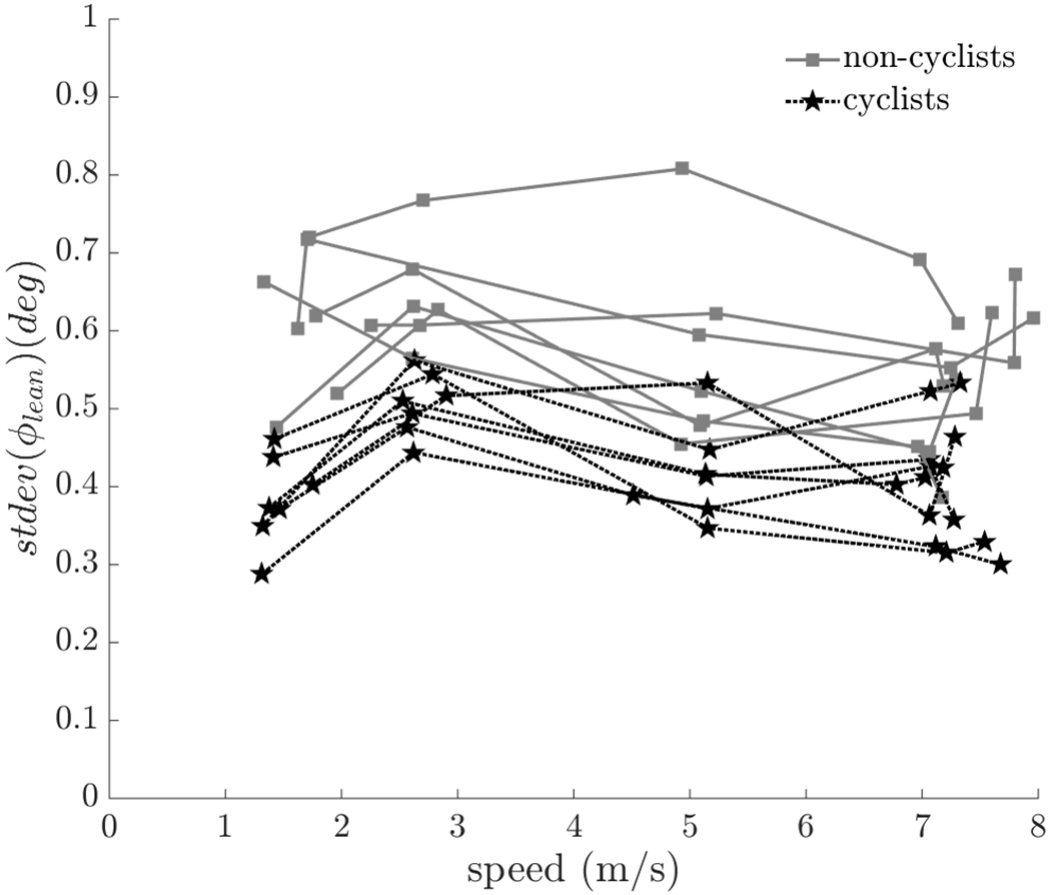
Standard deviation of rider lean angle (*ϕ*_*lean*_) versus speed. Cyclists exhibit significantly less rider lean than non-cyclists (F = 19.643, p < 0.001). All subject data are shown; connected points indicate data from the same subject.

In order to quantify rider lean variability, we report the standard deviation of the rider lean angle as a function of speed in Fig 12. Cyclists used less rider lean variation than non-cyclists (F = 19.643, p < 0.001) and riders used less rider lean variation as speed increased (F = 4.885, p = 0.031). The results suggest that all riders used smaller lean variation at higher speeds and that skilled riders (i.e. cyclists) used smaller lean variation overall to maintain balance.

### Differences between cyclists and non-cyclists

The results for cyclists and non-cyclists were similar at low speeds, but diverged as speed increased. At low speeds, all subjects commented that it was more difficult to maintain balance on the bicycle and to avoid riding off the rollers. At higher speeds, all subjects noted that riding on the rollers was easier. We believe that the best measure of balance performance is the cross-correlation of the lateral center of mass location (*y*_*COM*_) to the lateral center of pressure location (*y*_*COP*_). At low speeds, *R*^2^(*y*_*COP*,_ *y*_*COM*_) is similar between cyclists and non-cyclists, whereas at higher speeds *R*^2^(*y*_*COP*,_ *y*_*COM*_) is substantially less for non-cyclists.

To achieve better balance performance, cyclists used a different control strategy than non-cyclists. We quantified two types of control, *steering* and *rider lean*, by investigating the correlation of steer angle/rate and rider lean angle/rate to bicycle roll angle/rate. *Compared to non-cyclists*, *cyclists utilized greater rider lean than steering to maintain balance*, *especially at higher speeds*. Greater rider lean control decreased the need for additional steer control required to maintain balance. This conclusion is supported by the above observation that cyclists achieved better balance performance than non-cyclists despite exhibiting lower cross-correlation between steer and bicycle roll rates. To assess the relative importance of steer versus lean control on balance performance, we used a mixed linear model to address whether any measures of control (i.e., cross-correlations) predict the cross-correlation of *y*_*COM*_ to *y*_*COP*_, the adopted measure of balance performance. Again we used an alpha level of 5% and an auto regressive covariance model with order of one. The dependent variable was *R*^2^(*y*_*COP*,_ *y*_*COM*_) and the effects were *R*_2_(*ϕ*, *δ*), $R^{2}\left(\dot{\phi },\dot{\delta }\right)$, *R*^2^(*ϕ*, *ϕ*_*lean*_), and $R^{2}\left(\dot{\phi },{\dot{\phi }}_{lean}\right)$. Of the tested effects, only *R*^2^(*ϕ*, *ϕ*_*lean*_), the cross-correlation of rider lean to the bicycle roll angle, was a significant predictor of balance performance (F = 84.768, p < 0.001). Therefore, we conclude that rider lean was the dominant control strategy for balance performance for riding on rollers. The above results demonstrate that cyclists exploit rider lean control significantly more than non-cyclists to achieve better balance performance. Because the dynamics of a bicycle on rollers are similar to that of a bicycle overground [19], we do not expect that results for straight riding overground would be different than that results found in this study. Despite the importance of rider lean control on achieving better balance performance during straight path riding, maintaining balance using lean control is not better than using steer control, as steering has been demonstrated both experimentally and theoretically to be the dominant control method for stabilizing a bicycle [34].

**Fig 13:**
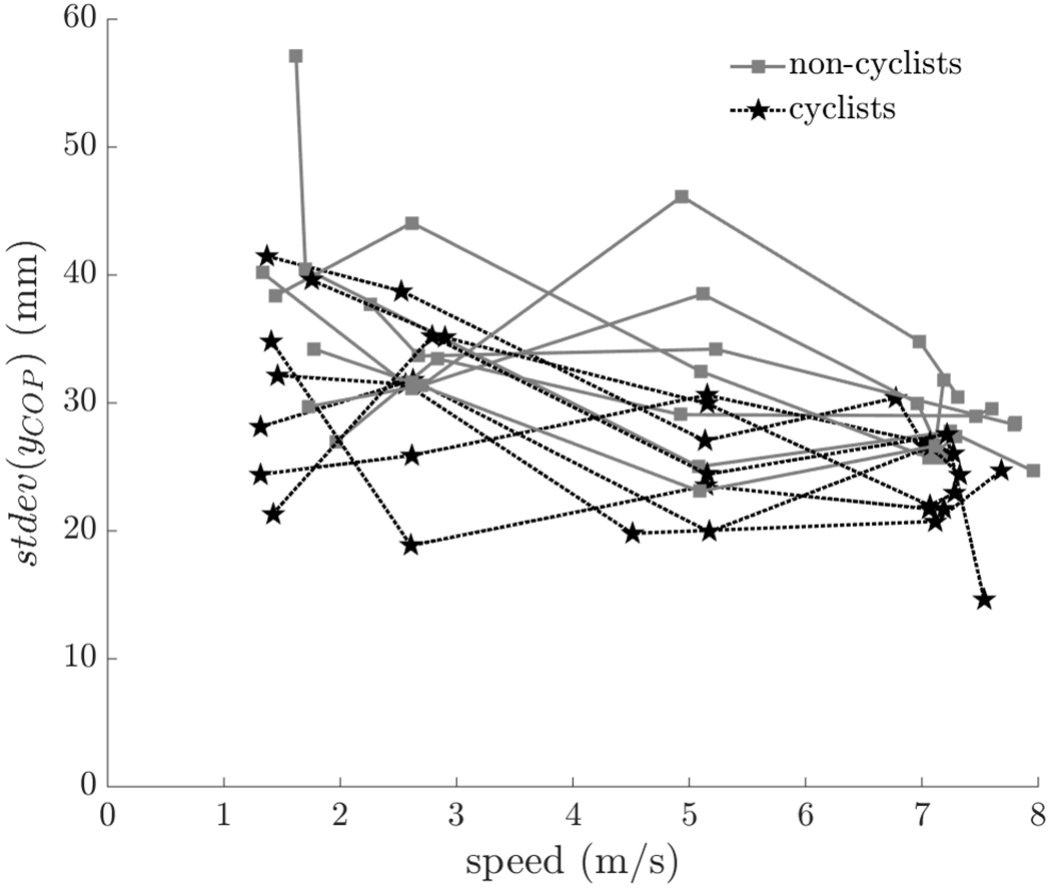
Standard deviation of the lateral position of the center of pressure (*y*_*COP*_) versus speed. The standard deviation of the lateral position of the center of pressure decreases significantly with increasing speed (F = 25.294, p < 0.001). Although it may appear that cyclists exhibit less variation in the center of pressure position than non-cyclists, there was not a significant difference between the two groups (F = 3.695, p = 0.059). All subject data are shown; connected points indicate data from the same subject.

In addition to achieving higher balance performance, cyclists also employ significantly smaller balance control inputs than non-cyclists. Cyclists and non-cyclists show similar variation in the lateral position of the center of pressure (i.e., lateral drift or side-to-side movement on the rollers) during riding (Fig 13). Thus, cyclists are not simply riding a significantly straighter path than non-cyclists. The similar movements of the center of pressure for cyclists and non-cyclists confirm that *both groups respond to essentially the same balancing task*. In other words, not only do cyclists use smaller control inputs to maintain balance, they use smaller control inputs despite facing the same balancing task as non-cyclists.

We have identified several metrics that distinguish rider skill in balancing, and hence stabilizing, a bicycle. Skilled riders exhibit higher correlation between the lateral positions of the center of mass and center of pressure, consistently use more rider lean than less skilled riders, and use significantly smaller control inputs than less skilled riders. While dynamic models of bicycles, such as the Whipple model [2], successfully predict the stability of an uncontrolled bicycle [35], they ignore the above stabilizing actions of the human rider. Metrics that define rider skill (such as those above) could reveal how bicycle design influences rider balance performance. For example, any bicycle designed to help riders maintain balance at low speeds should increase the cross-correlation between the lateral positions of the center of pressure and center of mass. Metrics of balance performance could similarly reveal the effectiveness of bicycle training programs. For example, riders with initially poor balancing skills could undergo a training program (perhaps riding a bicycle on training rollers, as shown in Fig 1) to accelerate skill learning. By quantifying differences between skilled and novice riders, we provide both researchers and practitioners with new tools to objectively assess the effects of different bicycle designs and training programs on real-life bicycle riding.

## Conclusions

Experimental results demonstrate that the best measure of balance performance is the cross-correlation between the lateral center of mass location (*y*_*COM*_) and the lateral center of pressure location (*y*_*COP*_). While *R*^2^(*y*_*COP*_, *y*_*COM*_) is similar between cyclists and non-cyclists at low speeds, it is substantially less for non-cyclists at higher speeds. Skilled riders use significantly smaller steering control input, exhibit less steering variation and less rider lean angle variation than novice riders. While riders exhibit similar balance performance at the slowest speed, at higher speeds skilled riders achieve superior balance performance by employing more rider lean control and less steer control compared to novice riders.

The metrics we introduce in this study lend themselves to quantifying balance performance of a bicycle/rider system for analyzing bicycle designs, assessing the performance of assistive technologies, and assessing beginner training programs. While some balance performance metrics rely on measures of the centers of mass and pressure that require sophisticated equipment, other metrics (such as those obtained from measures of angles and rates) may be readily obtained in practice using bicycle and rider-mounted inertial sensors (accelerometers and angular rate gyros) that detect steer angular velocity, bicycle roll angle, roll rate, and rider posture. Doing so in the future may be useful for evaluating rider adaptation to novel bicycle designs and quantifying learning during interventions that accelerate the acquisition of balance skill and/or the reduction of balance control inputs.

## Supporting Information

S1 Appendix Comparison of rider-bicycle COM calculation techniques. (PDF)

S1 Data Complete data set. The spreadsheet contains all metrics for each subject for each trial.
(XLS)
