# Supplementary material for: On the Skill of Balancing While Riding a Bicycle
Source: PLoS One. 2016 Feb 24;11(2):e0149340. doi: 10.1371/journal.pone.0149340 (PMC4766243; doi:10.1371/journal.pone.0149340)
Supplement: S1 Appendix — (PDF) [file pone.0149340.s001.pdf]

Appendix: Comparison of rider-bicycle COM calculation techniques

To our knowledge, the zero-point-to-zero-point integration technique has not been applied to study center of mass location during cycling. Therefore, we conducted four trials in which we compared the lateral location of the center of mass calculated from force platform data using the zero-point-to-zero-point integration technique (y_{COMfp}) with that calculated using extensive motion capture data (y_{COMmc}). The kinematic method utilized a 14-segment model to estimate the total body center of mass (1). The motion capture system tracks markers attached to each body segment and markers attached to the bicycle frame. Using the known location of the bicycle center of mass relative to the bicycle mounted markers (described in the appendix of (2)) and assumed mass fractions for each body segment (1), we calculated the center of mass location for the bicycle/rider system.

Similar to (3) and (4), we compared the center of mass location estimated using kinematic data (y_{COMmc}) to that estimated using kinetic data (y_{COMfp}) by calculating: the standard deviation (or root mean square) of each estimate (3), the standard deviation (or root mean square) of the difference between the estimates (3, 4), and the R^2 correlation between the estimates (3). In addition, we also calculated the slope of the least squares linear fit of y_{COMfp} to y_{COMmc}. In an attempt to quantify the accuracy of the kinematic method estimate of the center of mass location, we numerically differentiated the center of mass lateral position twice to obtain the lateral acceleration of the center of mass ($\ddot{y}_{COMmc}$) and compared the result to the lateral acceleration calculated from the force platform data ($\ddot{y}_{COMfp}$, measured lateral force divided by the mass of the rider-bicycle system). Similar to the center of mass comparisons, we compared the signals by calculating the R^2 correlation and the slope of the least squares linear fit of $\ddot{y}_{COMmc}$ to $\ddot{y}_{COMfp}$. Table 1 summarizes the results of these comparisons.

Table 1. Comparison of kinematic-based and kinetics-based estimates of center of mass location and acceleration.

trial	std(y_{COMmc})	std(y_{COMfp})	std(y_{COMmc} - y_{COMfp})	R^2(y_{COMmc}, y_{COMfp})	slope (y_{COMmc},y_{COMfp})	R^2($\ddot{y}_{COMfp}$,$\ddot{y}_{COMmc}$)	slope ($\ddot{y}_{COMfp}$,$\ddot{y}_{COMmc}$)
1	16.8 mm	26.2 mm	13.0 mm	0.825	1.41	0.387	0.36
2	17.0 mm	24.7 mm	10.5 mm	0.881	1.37	0.461	0.45
3	22.3 mm	31.5 mm	11.9 mm	0.921	1.36	0.574	0.66
4	18.1 mm	26.1 mm	10.2 mm	0.917	1.38	0.705	0.60

Similar to the findings of (3), we find that y_{COMfp} is highly correlated to y_{COMmc}. In (3), the authors reported cross-correlations (R) between the kinetics-based method to the kinematic-based method ranging from 0.79 to 0.96, which translate into R^2 correlation values between 0.62 and 0.92. Therefore, the cross-correlation results indicate that utilizing the zero-point-to-zero-point integration technique for bicycle riding seems to be as good if not better than using the technique for postural analysis. As evidenced by the standard deviations of y_{COMmc} and y_{COMfp} and the slope of the linear fit of y_{COMfp} to y_{COMmc}, the kinetics-based approach results in estimates of y_{COM} approximately 40% greater than the kinematic-based approach, with an RMS difference of about 11mm; these results suggest that perhaps using the zero-point-to-zero-point integration technique introduces some error due to the assumption that y_{COMfp} is equal to y_{COP} when the lateral force is zero. However, there is a low correlation of $\ddot{y}_{COMmc}$ to $\ddot{y}_{COMfp}$ and $\ddot{y}_{COMmc}$ is approximately 50% less than $\ddot{y}_{COMfp}$; these results suggest that using a kinematic-based approach may also be inaccurate. One explanation is that modeling the bicycle/rider as a system of rigid links does not allow small but important motions to be measured accurately during bicycle riding, which results in errors in y_{COMmc}. Using a kinematic-based approach may be inaccurate in

arriving at the acceleration of the mass center due to errors introduced in successive differentiations. For our application, we believe the kinetics-based method is superior because it is not sensitive to errors in marker placement, errors in modeling the body, and errors in body segment parameters.

References

1. Winter DA, Patla AE, Prince F, Ishac M, Gielo-Perczak K. Stiffness control of balance in quiet standing. Journal of Neurophysiology. 1998 September 1, 1998;80(3):1211-21.

2. Cain SM, Perkins NC. Comparison of experimental data to a model for bicycle steady-state turning. Vehicle Syst Dyn. 2012;50(8):1341-64.

3. Zatsiorsky VM, King DL. An algorithm for determining gravity line location from posturographic recordings. Journal of Biomechanics. 1998;31(2):161-4.

4. Lafond D, Duarte M, Prince F. Comparison of three methods to estimate the center of mass during balance assessment. Journal of Biomechanics. 2004;37(9):1421-6.
